# Usage of personas in the construction industry

**DOI:** 10.12688/openreseurope.15061.1

**Published:** 2023-02-16

**Authors:** Fausto Javier Sainz Salces, Marja Liinasuo, David Martin-Moncunill

**Affiliations:** 1 Escuela Superior de Ingeniería y Tecnología (ESIT), Universidad Internacional de La Rioja, Logroño, La Rioja, 26006, Spain; 2 Human factors, VTT Technical Research Center, Espoo, 02150, Finland; 3 Facultad de Tecnología y Ciencia, Universidad Camilo José Cela, Villafranca, Madrid, 28692, Spain

**Keywords:** Personas, Case study, Software design, User-centric design.

## Abstract

**Background:** This study on the use of personas was carried out during a building trade related software development EU project. Participants were introduced to the personas tool and encouraged to use it in their work in technical development.

**Methods:** A questionnaire was delivered three months after presentation and explanation of the remote tool, inquiring about benefits, drawbacks, intention of using personas in the future, and the role of usage related information as a whole in technical development.

**Results:** Previous knowledge about the tool, perceptions of the tool and its actual use seemed to influence developers in their decision to use it or not.

**Conclusions:** Almost half of the organizations in our study use real user-centric design methods in their technical development; this can ease the path for wider adoption of the methods when needed. One possible solution to strengthen the understanding and adoption of the user-centric methods is to spread information among technical developers, by identifying and using efficient communication channels, such as tailored training. User-centric methods seem to have gained followers in the technical development arena, indicating that this may be a feasible way for further adoption of user centricity in product development.

## Plain language summary

The article presents the results from an exploratory study in which software developers used the persona tool to help them in the design and development of software for the construction industry. The persona tool tries to provide relevant information about the tool user needs, and in this way helps in deciding the functionality the software program should have. Results showed software developers were interested in the tool, but its use was limited.

## Introduction

A persona, a fictitious person representing a group of people, was first introduced to the design community in the late 90’s ( Cooper, 1999). Personas have been used as a design tool for many years since then to foster ideation and empathy with different user groups. The method originally appeared in the context of software development and has become a widespread method adapted to many design disciplines and processes, such as IT systems or product development, user experience design, service design, communication strategy, and even marketing ( Bradley *et al.*, 2021; Nielsen & Hansen, 2014; Pruitt & Grudin, 2003). Personas are often considered as a method of promoting usability, but it can have a broader focus than this, providing information of, for example, areas of interest of the user, affecting the content of the IT application, system, or a product ( Nielsen, 2019).

We studied the usage of personas in SPHERE, an EU project aiming to provide a building information model (BIM)-based digital twin platform to optimize the building lifecycle, reduce costs, and improve energy efficiency in residential buildings. Several IT applications for professional use are being developed in the project, to be connected via the platform. Most of the project parties are technological by nature, but in the project user-centered design approach is also included. The project utilizes user-centric design methods, but personas are excluded. We wanted to find out whether this relatively usual and long-known method is used among technical developers in our building-trade centered project. We also presented the persona concept to project parties so that it also provided an opportunity to become acquainted with the concept and enabling its usage in the SPHERE (or other) project. As a research activity inside the project, we received approval from the Consortium and Project coordinator to carry on the study. Participants consented to the study (reading documentation and answering a survey) as it was also part of the work carried out during the project.

In the next section, Scientific background, we present briefly what is meant by personas in scientific literature; in Methods, we describe how our study was conducted and present our research; in Results, the results are presented briefly; in Discussion we contemplate the results as such and also relative to scientific literature; and in Conclusions, we provide the conclusions.

## Scientific background

Personas are typically used in an early phase of a design process to introduce the future users of the design artifact, with their needs and aspirations ( Bowen *et al.*, 2020), but they can be used throughout the development process to ensure that users’ critical needs and requirements will not be neglected. Personas can be used in various ways and phases in a project.

Regarding the basis for persona creation, different origins can be identified. In a simple form, persona can be, for example, a totally fiction-based collection of features, combined to a brief persona description, to foster ideation in the planning phase of a project. Persona can also be represented by a sketchy description of some person type, based on a couple of interviews, for briefly illustrating some specific role in research results. Persona creation can also be more time-consuming, utilizing field data collected through interviews, observations and questionnaires ( Huynh *et al.*, 2021). Then, the persona description can include, for example, computer skills, market size and influence, activities of a typical day, fears, and aspirations ( Nielsen, 2007). Personas can also be created automatically, such as producing them with YouTube Analytics data ( Salminen *et al.*, 2019). The usage of such data-driven, the so-called automatic persona generation, originating personas can be useful for the acceptance and supposed objectivity of the method, but care is needed in also this type of persona design. Demographic bias (bias in age, gender, or country representation) in algorithmic decision-making can take place with a smaller number of personas – for example, in the study of Salminen *et al.* (2019), the smaller number of personas created automatically resulted in underrepresentation of female personas.

The usual presentation of personas is a static one – a written description of persona features is provided to persona users, with a drawn figure or photo of some anonymous person. Also, new ways to present persona have been developed. Bonnardel and Pichot (2020) created an avatar with anticipated future user qualities and let participants to interact with the avatar in a virtual world. The results with these so-called dynamic personas (as presented by Bonnardel & Pichot [2020]) were statistically different from the ones obtained with static personas and proved to be worth developing further. 

With technology development and cumulating insight about the possibilities the personas provide, new ways to use personas will probably continue emerging.

Based on literature, personas are classified into four different perspectives: the goal-directed perspective, the role-based perspective, the engaging perspective, and the fiction-based perspective ( Nielsen, 2019). Essential in the goal-directed persona is that the (work) goals of the persona are the focus in the persona description. The role-based perspective shares goals and the focus on behavior of the goal-directed perspective but includes both qualitative and quantitative qualities, enabling persona to contain information not only about individual-related information (activities, hopes and fears) but also of market share, focusing on the persona’s role in the organization. The engaging perspective has its purpose in creating involvement and insight of user qualities for technical developers. Fiction-based perspective can be used for exploring design and generate discussion. For instance, extreme characters help in exploring the edges of the design space ( Djajadiningrat *et al.*, 2000). All persona types except for the last perspective are based on user data. Thus, personas can also be described by at least three dimensions: the origin of the features included in the description, the deepness and variety in which personas are represented, and by the purpose of them. Naturally, the purpose for the usage of personas should dictate the decisions made regarding the other dimensions.

## Methods

### Participants

We contacted the whole consortium, i.e., members of 18 organizations in SPHERE project. Of the 18 companies conducting technical development in the project, 13 companies were the ones to which the study was really targeted, as software developers that could use the Persona tool if they wanted to do so. We received 11 responses from 10 companies. All 11 respondents were technical developers, including the managerial responsibilities of three respondents.

### Questionnaire

The questionnaire ( Liinasuo & Sainz, 2023) included questions about: (i) the background of the respondent (2 questions), (ii) experience with the personas method (5 questions), (iii) opinions about personas as a method (7 questions), and finally (iv) the usage of other user related methods in technical development (1 question).

### Proceeding of the study

We sent an information package of personas to the consortium of the project, with a general-level description of what is meant by personas along with five sample personas representing professionals in the building trade. To briefly describe the content of the information provided, personas were defined as typical users whose goals and characteristics represent the needs of a larger groups, clarifying what the user wants. The content of the information was in a rather abstract level, not pinpointing the benefits nor weaknesses of the method, and neutral in the sense that the responder was not expected to start using the method. The delivery of future query about the subject was not mentioned in the information package. After two weeks we sent the questionnaire. Reminder messages were sent to organizations which conducted technical development and had not responded, with the first reminder being sent after one month. Responses were sent within four months, with the first answer received on the same day as the questionnaire was sent.

### Data analysis

Data analysis was performed by clustering the responses according to the respondents’ previous knowledge and experience with personas as a method. The number of respondents is rather small, and many responses were also missing (26 missing responses and 7 misunderstood questions with inappropriate responses to the questions about the opinions of personas and the usage of other user related methods, the total of the possible responses to these themes being 88 responses). Hence, all individual responses could be reported in this paper, with only some shortening.

### Research questions

Our research questions are the following:

1. Are technical developers using personas in their work?
2. Why do technical developers use personas / do not use personas?
3. Are other user-centric methods used in technical development?

## Results

Regarding experience with personas, two participants had used personas before the SPHERE project but neither of them had used personas in the project. One participant became familiar with personas in the project, based on the information package, and had used it in technical development ( Liinasuo & Sainz, 2022). One participant considered personas the same as user types but had never used the method personally. Thus, there were four participants altogether who were familiar with personas in some way, and seven participants who were not familiar with the concept before the project and did not use it in the project either. In the following section, results are classified according to the previous knowledge and experience regarding personas, taken for granted that all participants have at least received the information package about personas in the current project.

Participants were asked in what phase it would be beneficial to use personas – in the beginning, at the middle or at the end of the design process. One participant who had used personas before the current project considered personas useful in the beginning, after the decision about the vision and main functionality of the tool, to let user needs affect design and detailed functionalities of the tool, and at the end of design for marketing purposes. Another participant, who also had used personas only at earlier stages of the process, thought that personas should be used in the beginning, because before starting to build the tool you should know who you are doing it for. One participant, who had used personas in the current project only, thought that personas should be used in all project phases – first to ensure that all relevant aspects in tool’s operation are taken into account, in the middle to avoid losing sight of the objectives set by users, and in a way also at the end as then. They felt that it must be ensured that design has not gone astray and, if it has happened, the related problems need to be solved. One participant with earlier knowledge of personas, but without experience with personas, considered that personas should be used only in the middle of the project, to ensure that the design works with users with different needs, such as color blindness.

Without prior knowledge nor experience, one participant found personas useful in the beginning of the design, to have user needs in mind, and at the end of the design, to check the best personas suitable for the tool. Two participants thought that personas are useful only in the beginning of the design process for taking user requirements into account. Another participant considered personas the most useful in the middle of design to enable user needs affect design through the iterative phases of technical development. One participant thought that personas are needed in the beginning to keep technical development on the right track and to avoid mistakes, in the middle to ensure that development proceeds to right direction, and at the end to see whether the end result is in accordance with the needs of personas.

Participants were asked about benefits and weaknesses of personas. Regarding benefits, it was contemplated that the usage of personas supports developer, product owner, designer, marketing and salespeople in aligning their actions to the target group, makes the target group clearer and sometimes makes it easier to make decisions, and makes it easier to discuss with investors and other shareholders as the concrete examples personas provide make it easier to understand the target group (participant with earlier experience with personas), provide insight about the end user’s needs (participant with earlier experience with personas), support the developer to have a thought about who, when and why somebody would use it (participant who had used personas in current project only), and ensure that the solution is suitable for a wide range of users (participant with earlier knowledge about personas). The following opinions are provided by participants, who had no prior knowledge or experience about personas; the benefit in using personas is that it puts effort in knowing needs and use cases of real users; allows thinking “out of the box”, that is, taking into account a point of view not having thought of before; with personas, the solution meets better the needs of the end users; and supports designers, architects and engineers in knowing users’ expectations and requirements.

Regarding weaknesses of personas, the complete target group may not be included in personas, and updating is necessary because target groups can change and the features of the product may also change over time (participant with earlier experience with personas), it is difficult to keep balance between generic and specific criteria for a persona (participant with earlier experience with personas), there are no contraindications to the use of personas as long as user profiles are made professionally and with experience (participant who had used personas in the current project only), and personas do not replace real people and thus provide only limited insight, and are mostly useful as facilitating conversations (participant with earlier knowledge only about personas). Participants without earlier experience or knowledge of personas responded as follows: there is the risk to be too generic or theoretic; opinions of real users are not necessarily reached; it is difficult to establish common mechanisms that are valid for all SPHERE tools, different from each other; and personas do not fit to all situations, such as automated solutions functioning in the background without the involvement of people, removing the need for a user interface.

Participants were also asked to reason why or why not to use personas in the future. The questions were separate from each other. Some responded to one of the questions (why personas are used in the future or why personas are not used in the future) and some contemplated both. In the following section, responses are combined according to the participant.

Personas would be good to be always used in bigger products because then it is a really useful tool to focus on right things, and maybe not in smaller projects because personas would cost too much time (participant who had earlier used personas). Personas could be used in the future because it helps to talk about the same point of view between different stakeholders (participant who had earlier used personas). Personas improve the effectiveness of the tool, and the use of personas explains why consumer goods such as clothing are designed differently depending on the geographical market area; the main reason for not using personas is the distorted profile of the design team, only focusing on technology (participant who had only used personas in the current project). It would be good to use personas in the future as it enables thinking from a wider user perspective, and when thinking why not to use personas; it is better to do real-life user testing instead of imagined personas (participant with earlier knowledge but no experience with personas). In creating models, lessons learned from similar projects are used, as well as feedback provided from other companies (response to the question of main reason(s) to use personas in the future, response from a participant without earlier knowledge or experience of personas). The main reason to use personas is that it improves the final product, and regarding why not to use personas, that it may not be necessary to use personas in each project due to different characteristics of each project (participant who had no earlier knowledge or experience on personas). Personas could be used because then the technical solution would better meet the needs of the end user (participant who had no earlier knowledge or experience on personas). Personas provide clear user types that everybody can align around, and it also helps in identifying the user we are designing for (participant who had no earlier knowledge or experience on personas).

Finally, it was asked whether some other user or usage centric method has been used in technical development. The responses of participants with earlier knowledge of experience with personas were as follows: workshops, questionnaires and interviews regarding prototypes and focus groups (participant who had earlier used personas); analysis of technological development of the target market, level of technical training of the people who are going to use the tool, equivalent solutions already in target market, aesthetic and functional preferences of potential users, level of economic development in target market regarding the selling price, all considered relevant regarding user profile, especially important to tool’ user interface (participant who had used personas in the current project); iterative internal and external user testing (participant who had also earlier known but not used personas).

The rest of the responses are from the participants without earlier knowledge or experience with personas. They are as follows: value proposition analysis, customer segmentation analysis with customers’ and users’ needs and wishes considered, usually from the exploitation or marketing points of view; carrying out workshops and/or real demonstration with end users and/or other stakeholders as well as collecting real opinions; previous experiences and background relationships with customers are taken into account; the Lean User Experience (UX) methodology; and user interface and functionalities are improved based on test users using the solution.

## Discussion

In the study, technical developers’ opinions and experiences with the persona method were probed with a questionnaire. Before the query, all 11 participants had received information and examples of personas as a method with which to improve user centricity in technical development on a voluntary basis. Four participants had used or known of the concept before the current project, and seven participants had only read the persona information – if at all.

Questionnaire responses are sensible, most of them emphasizing the importance of acquiring users’ opinions affecting design, improving the end result. The phase in design in which to use personas was not mentioned in the information package, so the opinions entirely originated from the participants’ own ideas. No participant considered the usage of personas only at the end of design, which shows that participants had understood at least some basic information about personas. Participants also provided insight about weaknesses in the method, a matter not presented in the information package; examples of such responses are that personas may not capture the real opinions of possible users, and it can be difficult to keep balance between generic and specific criteria for a persona. As a whole, participants seem to be in favor of the usage of personas. When asked about the reasons why to use or not use personas in the future, all responses except for one found reasons to use personas in the future. It must be borne in mind, though, that positive responses may also reflect participants’ (not necessarily conscious) willingness to please the questionnaire creators.

Some misunderstandings showed up, though. One participant considered that, when it is a question of automated solutions functioning as is if in the background, personas are not needed. This is not the case, personas can be used in this situation for indicating what kind of features in that background solution are preferred by users, for instance for telling suppliers what users value, to support product marketing. One participant had misunderstood that the same personas should serve the design of all building trade related tools. This cannot be the case as users’ needs vary, depending on the user’s profession and tool in question, and the same users hardly use all building related tools. One participant stated that solutions are better to be tested with real users instead of having personas, as if personas would substitute user testing.

Furthermore, one participant appeared to exaggerate the importance of personas, probably because the participant had just used the method in design for the first time, and was perhaps overexcited about the method. That participant may not be highly knowledgeable about the user centric design methodology as a whole, as according to that participant, not using personas shows that the design team has a distorted profile, focusing excessively on technology.

The responses to the question of the usage of other user centric methods shows that user related data is found important, but user centricity as conceived in human factors or the cognitive ergonomics approach is not necessarily a shared value or understood in technical development. The responses, including testing, workshops etc. with real users, which was mentioned by six participants, are well-representative of user-centricity. Responses including the consideration of technical training for people who are going to use the tool, customer segmentation analysis, and taking into account previous experience with customers, show that potential users are reflected on but not effectively. This can be considered as a good result as the competence of technical developers is in technology and it is hard to go beyond the boundaries of personal expertise.

The results present a sample of opinions and experiences of technical developers in current software design. The sample is not representative in any way, but it may provide a rough overview of the status of personas in technical design: they are not widely used and not very well known. There seems to be room for improvement in the understanding of user centric design methods, not to mention the benefit of such methods. However, the sophistication shown in many responses about persona related features also reflect the potential for more usage-centric design. Accordingly, participants seem to use real users during the design, which supports in designing such solutions which truly serve the users and are according to users’ preferences. Also, such participants, who did not identify truly usage centric methods, showed that designs cannot be made without the user in mind. Some information about the users must be acquired and personal impressions should not be solely counted on.

Thus, regarding our research questions, it can be stated that technical developers in the software construction industry are not using the persona tool much (the research question “Are technical developers using personas in their work”).

The reason for this is hard to conclude based on questionnaire responses (the research question “Why do technical developers use personas / do not use personas”). Many participants identified factors, which speak for the usage of personas. Benefits that appear in the responses are also presented by, for example, Tomlin (2018) who stated that personas reduce scope creep, that is, they support maintaining the scope of the design project. Most responses show that developers understood what personas are like as a usage-centric method ( Nielsen *et al.*, 2021). This roughly indicates that the reason for not using personas is something else than misunderstanding. It was feared that personas might not reflect real users (reflected in responses like “they [personas] do not replace real people and thus provide limited insights”, and “there could be the risk to be too generic or theoretic”). This could perhaps be because the creation of personas was considered difficult or cumbersome. The fact that only part of the participants reported about the usage of comparable usage-centric methods may be connected to the little use of personas (the research question “Are other user-centric methods used in technical development”) – based on the responses, technical development seems to be rather technically oriented. Still one possible reason is that user information is conveyed by other means, such as user requirements or the like, not written in questionnaire responses. This, in turn, may be because technical developers are not necessarily responsible for bringing users, directly or indirectly, to the technical project. Even if users’ needs may affect development, user related information is not focused on as this information is brought to developers by representatives of other professions.

Still one reason for the lack of interest in personas, as a response to the information package sent in the project, could be that the communication between technical developers and the human factors professionals in SPHERE project was not as fluid as desired, considering also the geographical constrains in a consortium where work is usually carried out remotely with meetings held at project members’ request. Thus, it seems that misunderstandings and communication problems can also be blamed, as pointed by Bonnardel and Pichot (2020) and considering some of the answers to the questionnaire. Furthermore, not teaching the personas in a face-to-face manner might be one of the reasons ( Nielsen, 2007) for the received feedback. It might be that, as stated by Blomquist and Arvola (2002), poor introduction to the method is the main cause of rejection of the personas tool or poor use, as well as a poor incorporation of the method in the design process ( Nielsen *et al.*, 2021). On the other hand, it is rather unlikely that a new method would be used based on just a presentation in a project. People tend to keep working practices unless it is shown that new ones are highly beneficial. It must also be taken into account that if performed well, the building of personas takes time as first the decision is needed in organizational level to invest in it, then people or person dedicated to persona building etc. – the decision to use personas is an investment which takes time, effort and money. Furthermore, the use of personas is one way to be user centric in technical development but there are also other means, which are also used in some of the participants’ companies (workshops, user testing etc.). Using personas is not the criterion of being user-centric in technical development.

Regarding the usage of the user-centric methods in technical development as a whole, it seems that in about half of the development projects represented in this study, developers and their colleagues’ contact users for direct questioning, testing and workshops. The other half is interested in the user in a more distant way. This is a good situation. There have been efforts to combine technology-driven and user-centric approaches for a long time (see, e.g., DeBellis & Haapala, 1995; Eriksson *et al.*, 2005; Ferreira *et al.*, 2018) and, based on our results, the user-centric perspective seems to slowly but gradually gain momentum.

## Conclusions

The persona tool was well received among our case study participants and, in accordance with this, as much as about half of participants used user-centric methods supporting technical development and the rest had more technically oriented approach, not excluding user or customer related information.

Our results show that we are on our way to increase the adoption of user-centric methods in design; however, it still takes time for the methods to become widely adopted. The use of personas is one possibility for those in technical areas to get closer to user centricity. There are several user-centric design methods, however, use of personas is not the only one, and it is impossible to define what the best one would be for any specific development. It depends on the features of the product to be developed and also on other factors, such as whether an entirely new product is in question, or whether the product is being built on something which has been used already, in which case some possible usability issues might have been identified already. One part of the solution can be a more effective delivery of information about the user-centric methods to technical developers. This study is an example showing that mere information is not enough; information delivery should be followed by more effective means, such as tailored training. This training could be connected with development work at hand, supporting the perceived meaningfulness of the method. It would be advisable that technical developers, not only human factors professionals possibly working in the companies, continue to adopt or at least familiarize themselves with user-centric design methods. That would strongly support the development of products which are even closer to users’ needs and requirements. Although all participants in the research project answered the survey, we understand that the size is small. However, as this is an exploratory study the information gathered is relevant considering the data sample and reduced population of software developers. Further research should consider extending the survey to the population that did not participate directly in the research project, but belongs to the participating organizations.

## Data Availability

Zenodo: SPHERE personas research. https://doi.org/10.5281/zenodo.7249706 ( Liinasuo & Sainz, 2022) This project contains the following underlying data: Personas_Survey_Results.zip Zenodo: SPHERE Personas survey. https://doi.org/10.5281/zenodo.7503769 ( Liinasuo & Sainz, 2023) This project contains the following extended data: OpenResearchSPHERE Personas survey_April2021.docx (blank survey document) Data are available under the terms of the Creative Commons Attribution 4.0 International license (CC-BY 4.0).
