# Supplementary material for: Usage of personas in the construction industry
Source: Open Res Eur. 2025 Apr 10;3:33. Originally published 2023 Feb 16. [Version 2] doi: 10.12688/openreseurope.15061.2 (PMC12009476; doi:10.12688/openreseurope.15061.2)
Supplement: Peer review history

# Reviewer report: Reviewer response for version 2

**DOI:** 10.21956/openreseurope.18210.r53048

**Published:** 2025-04-24

**Authors:** Lene Nielsen

**Affiliations:** 1 IT University of Copenhagen, Copenhagen, Denmark

No further comments

Is the study design appropriate and does the work have academic merit?

Partly

Is the work clearly and accurately presented and does it cite the current literature?

Partly

If applicable, is the statistical analysis and its interpretation appropriate?

No

Are all the source data underlying the results available to ensure full reproducibility?

No

Are the conclusions drawn adequately supported by the results?

No

Are sufficient details of methods and analysis provided to allow replication by others?

Partly

Reviewer Expertise:

Personas

I confirm that I have read this submission and believe that I have an appropriate level of expertise to confirm that it is of an acceptable scientific standard.

# Reviewer report: Reviewer response for version 2

**DOI:** 10.21956/openreseurope.18210.r53050

**Published:** 2025-04-19

**Authors:** Sylvain Fleury

**Affiliations:** 1 Ecole Nationale des Arts et Métiers, Paris, France

No further comments to make

Is the study design appropriate and does the work have academic merit?

Partly

Is the work clearly and accurately presented and does it cite the current literature?

Yes

If applicable, is the statistical analysis and its interpretation appropriate?

Not applicable

Are all the source data underlying the results available to ensure full reproducibility?

Yes

Are the conclusions drawn adequately supported by the results?

Yes

Are sufficient details of methods and analysis provided to allow replication by others?

Yes

Reviewer Expertise:

Cognitive ergonomics

I confirm that I have read this submission and believe that I have an appropriate level of expertise to confirm that it is of an acceptable scientific standard.

# Reviewer report: Reviewer response for version 1

**DOI:** 10.21956/openreseurope.16285.r32228

**Published:** 2023-10-16

**Authors:** Beenish Moalla Chaudhry, Ashraful Islam

**Affiliations:** 1 Computer Science and Engineering, Independent University, Dhaka, Dhaka Division, Bangladesh; 2 University of Louisiana at Lafayette, Lafayette, Louisiana, USA

The paper explores the utilization of personas, a design tool representing groups of people, in software development and other design disciplines. It focuses on their usage in a project aimed at creating a digital twin platform for residential buildings. The study investigates whether personas are used by technical developers in the building trade industry and examines the benefits, drawbacks, and future intentions regarding their usage. The findings indicate that personas can be valuable for technical developers in fostering user-centricity and empathy, influencing the content of IT applications and systems. The paper emphasizes the importance of spreading information about user-centric methods among technical developers through tailored training and efficient communication channels. Overall, the research suggests that incorporating personas and other user-centric design methods can have practical implications, enabling technical developers to better understand user needs and create more user-friendly products and systems. The study also highlights the influence of previous knowledge and perceptions of personas on their adoption among developers, with about half of the organizations using user-centric methods.

 The results of the paper show that previous knowledge about personas, perceptions of the tool, and its actual use seemed to influence developers in their decision to use it or not. Out of the eleven participants in the study, four were familiar with personas in some way, and seven were not familiar with the concept before the project and did not use it in the project either. One participant became familiar with personas in the project, based on the information package, and used it in technical development. The paper concludes that almost half of the organizations in the study use real user-centric design methods in their technical development, which can ease the path for wider adoption of the methods when needed. The study also suggests that spreading information among technical developers about user-centric methods, by identifying and using efficient communication channels, such as tailored training, can help strengthen the understanding and adoption of these methods.

**However, the limitations of this paper are:**

 The sample size of the study is relatively small, with only eleven participants, which may limit the generalizability of the findings. The study was conducted in the context of a specific building trade-related software development EU project, which may limit the applicability of the findings to other contexts. The study relied on self-reported data from participants, which may be subject to bias or inaccuracies. The study did not compare the effectiveness of personas to other user-centric design methods, which could provide a more comprehensive understanding of the benefits and drawbacks of different approaches. The study did not explore the long-term impact of using personas on the development process or the quality of the final product.

 Although the paper is well-written, well-formatted, and clearly understandable, the following are my concerns regarding this work:

1. The title doesn’t fully and appropriately support the experiment shown in the paper. The title tells about the construction industry but I couldn’t see such contributions toward the construction industry rather than the technical developers.
2. There is not sufficient correlation presented among the research questions, the methods, and the results. For example, RQ 2 states that “Why do technical developers use personas / do not use personas?” but there is no relationship explained between this RQ and the aim of the work to answer why this RQ is necessary.

Is the study design appropriate and does the work have academic merit?

Yes

Is the work clearly and accurately presented and does it cite the current literature?

Yes

If applicable, is the statistical analysis and its interpretation appropriate?

Not applicable

Are all the source data underlying the results available to ensure full reproducibility?

Yes

Are the conclusions drawn adequately supported by the results?

Yes

Are sufficient details of methods and analysis provided to allow replication by others?

Yes

Reviewer Expertise:

Human-Computer Interaction, Usability, User Experience

We confirm that we have read this submission and believe that we have an appropriate level of expertise to confirm that it is of an acceptable scientific standard, however we have significant reservations, as outlined above.

# Reviewer report: Reviewer response for version 1

**DOI:** 10.21956/openreseurope.16285.r33666

**Published:** 2023-08-10

**Authors:** Farshid Anvari

**Affiliations:** 1 Minh Hien Pty Ltd., New York, NY, USA

In this paper, the authors surveyed developers of tools for construction industry about their usage of personas during development work. The authors receive 11 responses. Based on their analysis of the report the authors concluded that software developers were interested.

 The paper suffers from numerous flaws and cannot be accepted as a scientific paper. The literature gap is not identified. The research questions are unclear. The authors do not answer why they selected the industry and how they selected the participants for the survey.

 The paper lacks proper data or statistical analysis.

 The paper structure is poor and does not follow the research paper norm.

Is the study design appropriate and does the work have academic merit?

No

Is the work clearly and accurately presented and does it cite the current literature?

Partly

If applicable, is the statistical analysis and its interpretation appropriate?

No

Are all the source data underlying the results available to ensure full reproducibility?

Yes

Are the conclusions drawn adequately supported by the results?

Partly

Are sufficient details of methods and analysis provided to allow replication by others?

Yes

Reviewer Expertise:

I have authored and used persona for research purposes.

I confirm that I have read this submission and believe that I have an appropriate level of expertise to state that I do not consider it to be of an acceptable scientific standard, for reasons outlined above.

# Reviewer report: Reviewer response for version 1

**DOI:** 10.21956/openreseurope.16285.r33663

**Published:** 2023-08-09

**Authors:** Devi Karolita

**Affiliations:** 1 Monash University, Melbourne, Australia

Thank you for sharing your paper. Here are my comments:

 The **title** of the paper struck me as misleading, as I initially anticipated an exploration of persona usage within the construction industry. Regrettably, this isn't the case.

 While the **Abstract** specifies the study's domain (technical development) and participants (technical developers), these terms lack a formal definition in the body of the paper.

 Several sections, notably the **Introduction** and **Scientific** **Background**, require proper citations. For instance, the statement "Personas have been used as a design tool for many years since then to foster ideation and empathy with different user groups." (p.3) and "... but they can be used throughout the development process to ensure that users’ critical needs and requirements will not be neglected." (p.3) demand citations. Additionally, an illustrative persona example could enhance readers' grasp of the concept.

 In the **Methods** section, the paper indicates that participants' responses were subjected to a clustering approach for analysis. Strangely, the presentation of results leans more towards a qualitative approach.

 Turning to the **Results**, there are a few observations. First, rephrasing the research questions could align them more effectively with the ensuing Results. For instance, rephrasing RQ#1 to "Which phases are personas employed in?" could improve cohesion.

 Second, the section would benefit from examples illustrating the beginning, middle, and end of design-related tasks.

 Third, addressing each RQ appears somewhat convoluted. For instance, the explanation of RQ2 is challenging to follow and seems to lack continuity (p.5, paragraph 3). Furthermore, the example provided (consumer goods like clothing) seems misplaced within the context of the paper (construction industry).

 Lastly, elaborating on the rationale behind participants' integration of user-centric methods in their tasks could enhance the answer to RQ3.

 Comparable to the Results section, the **Discussion** section's coherence is also a bit muddled. To aid readers' comprehension, consider introducing subsections in both these sections to serve as guideposts throughout the paper.

Is the study design appropriate and does the work have academic merit?

Partly

Is the work clearly and accurately presented and does it cite the current literature?

Partly

If applicable, is the statistical analysis and its interpretation appropriate?

No

Are all the source data underlying the results available to ensure full reproducibility?

No

Are the conclusions drawn adequately supported by the results?

No

Are sufficient details of methods and analysis provided to allow replication by others?

No

Reviewer Expertise:

Personas, requirements engineering

I confirm that I have read this submission and believe that I have an appropriate level of expertise to state that I do not consider it to be of an acceptable scientific standard, for reasons outlined above.

# Reviewer report: Reviewer response for version 1

**DOI:** 10.21956/openreseurope.16285.r33661

**Published:** 2023-07-28

**Authors:** Lene Nielsen

**Affiliations:** 1 IT University of Copenhagen, Copenhagen, Denmark

The paper reports on a trial of introducing personas for technical development in the construction industry. The data is collected from a questionaire.

 The result shows that previous knowledge of the method influences use.

 Unfortunately the paper has several flaws in relation to scientific rigor and report.

 1) It is difficult to understand how this particular group of software developers are different from other software developers - the title "Usage of personas in the construction industry" is thus giving a false impression that it is used in the building industry, while the use is more accurately in software development for the building industry.

 2) The personas tool is not described in detail, thus it becomes difficult to understand the case.

 3) The questionaires include mainly open answers, but the analysis does not include quotes, thus it becomes difficult to access the result section. Furthermore

 the questionaire has focus on previous knowledge and use of personas and not the specific case.

 4) Most problematic is: The research questions are presented as:

      1. Are technical developers using personas in their work?

      2. Why do technical developers use personas / do not use personas?

      3. Are other user-centric methods used in technical development?  

 - with a questionaire with 11 answers it becomes difficult to draw any conclusions that is so generic to include all developers.

 5) The result section is very difficult to read - it would benefit from a table that shows the results and the difference between those who have used personas before and those who have not. One example of how difficult it is to read is a sentence of 13 lines! It could be beneficial to do a text analysis of the answers and treat the data as qualitative data, this might provide some interesting results.

 6) The conclusion of: "The sample is not representative in any way, but it may provide a rough overview of the status of personas in technical design: they are not widely used and not very well known."

 The small sample makes it difficult to conclude anything, especially given that personas is mainly used in the UX design phase.

 7) The discussion of: "Still one reason for the lack of interest in personas, as a response to the information package sent in the project, could be that the communication between technical developers and the human factors professionals in SPHERE project was not as fluid as desired," points to the problem of what it is that is researched in this paper - is it the case or the use of personas in general?

 8) The conclusion that "This study is an example showing that mere information is not enough; information delivery should be followed by more

 effective means, such as tailored training." is an example of the problem of asking about general knowledge of the method and then concluding in general. There are few questions about the specific use of personas in the specific case, but most are on general knowledge and use, so the question is: what can the authors conclude?

 9) The paper has a number of language errors.

 10) To move forward and resubmit the authors could consider to both look at previous experience and create a table for that and to look at if the role of the participants has any influence. To include quotes from the survey in the results. To include an illustration of the persona tool and describe the tool in detail. To consider the literature of when in the design process personas are used e.g. the works of Miaskiewicz and Kozar (2011 ^1^) and Friess (2012 ^2^).

Is the study design appropriate and does the work have academic merit?

Partly

Is the work clearly and accurately presented and does it cite the current literature?

Partly

If applicable, is the statistical analysis and its interpretation appropriate?

No

Are all the source data underlying the results available to ensure full reproducibility?

No

Are the conclusions drawn adequately supported by the results?

No

Are sufficient details of methods and analysis provided to allow replication by others?

Partly

Reviewer Expertise:

Personas

I confirm that I have read this submission and believe that I have an appropriate level of expertise to state that I do not consider it to be of an acceptable scientific standard, for reasons outlined above.

## References

[1] : Personas and user-centered design: How can personas benefit product design processes?. *Design Studies* .2011;32(5) : 10.1016/j.destud.2011.03.003 417-430 10.1016/j.destud.2011.03.003

[2] : Personas and decision making in the design process: an ethnographic case study. *Proceedings of the SIGCHI conference on human factors in computing systems* .2012;1209-1218 Reference source

# Reviewer report: Reviewer response for version 1

**DOI:** 10.21956/openreseurope.16285.r32224

**Published:** 2023-07-27

**Authors:** Panca O. Hadi Putra

**Affiliations:** 1 Universitas Indonesia, Depok, West Java, Indonesia

Thanks for sharing your research. The following are my comments:

1. Abstract section: While you already mentioned data collection technique through a questionnaire, it is unclear what method you used to analyze the data. In addition, you wrote about user-centric methods in the conclusions of the abstract. Were you referring to the personal as a user-centric method? Please clarify this for your reader.
2. The use of persona in construction industry’s software development project is an interesting research topic. However, it is unclear from the introduction section what motivated you to conduct this research. What was the issue being experienced by the SPHERE teams?
3. Please also clarify the objective of this research in the introduction section. “We wanted to find out whether this relatively usual and long-known method is used among technical developers in our building-trade centered project" - this contradicts your previous statement stating that persona was not used in the project: “The project utilizes user-centric design methods, but personas are excluded”. The title of the manuscript should also reflect the objective the research.
4. Methods: Why did you only target 13 out of 18 companies in the project? Who are the units of analysis of this research? The software developers only? Please clarify. What was your sampling technique? How many people represent the population? How did you ensure that you meet the minimum sample?
5. Data analysis: What do you mean by “clustering the responses”? What method did you use to cluster the information?
6. Results: Is it possible to visualize the results and include descriptive statistics? Please ensure that all three research questions have been answered in the discussion section.

Is the study design appropriate and does the work have academic merit?

Yes

Is the work clearly and accurately presented and does it cite the current literature?

Partly

If applicable, is the statistical analysis and its interpretation appropriate?

Partly

Are all the source data underlying the results available to ensure full reproducibility?

Yes

Are the conclusions drawn adequately supported by the results?

Partly

Are sufficient details of methods and analysis provided to allow replication by others?

Partly

Reviewer Expertise:

User experience

I confirm that I have read this submission and believe that I have an appropriate level of expertise to confirm that it is of an acceptable scientific standard, however I have significant reservations, as outlined above.

# Reviewer report: Reviewer response for version 1

**DOI:** 10.21956/openreseurope.16285.r32229

**Published:** 2023-06-08

**Authors:** Sylvain Fleury

**Affiliations:** 1 Ecole Nationale des Arts et Métiers, Paris, France

This article describes a research in which technical developers on a European project were trained to the personas method, then later questioned by questionnaire to find out if they had used it, if so why and if not, why not.

 Generally speaking, the article seems well written and clear. The state of the art is interesting and well structured.

 My main problem is that I don't think the article's main contribution (which you find out about at the end) corresponds well to the angle chosen in the problem and the title. In my opinion, when you read the analysis of the results and the conclusions, you see that the article's contribution is in fact a reflection on the way in which specialists in the human factor can support developers in large collaborative projects. We can see that the package passed on to developers has not been very effective, and we can try to understand why and recommend ways of doing things that would be more relevant.

 The package given to developers at the start should be discussed more, because it seems to be an essential point. In my opinion, the title of the article is inappropriate. There's nothing specific to the construction sector in the article. Can’t we assume that the work can be generalized to technical developers in general? If the article needs to be reworked, here are my suggestions:

- Restate the title and the problem: the article's contribution is to reflect on how to support developers in user-centered design methods.
- “ *Most of the project parties are technological by nature, but in the project user-centered design approach is also in-cluded. The project utilizes user-centric design methods, but personas are excluded. "* : This part is convoluted and unclear: what was the overall organization of the project, and then what were its specificities?
- “ *Participants consent*": specify whether this was verbal or whether they signed something.
- *"Personas can be used in various ways and phases in a project.*”: This sentence doesn't add anything to the previous ones, can it be deleted?
- In participant section: Briefly explain why those can use personas and not the others? Because these are HCI developments, whereas the others only developed back-end?
- The number of missing responses appears to be high. Maybe the authors have to explain why.
- It seems to me that research question should be before "Methods" section.

Is the study design appropriate and does the work have academic merit?

Partly

Is the work clearly and accurately presented and does it cite the current literature?

Yes

If applicable, is the statistical analysis and its interpretation appropriate?

Not applicable

Are all the source data underlying the results available to ensure full reproducibility?

Yes

Are the conclusions drawn adequately supported by the results?

Yes

Are sufficient details of methods and analysis provided to allow replication by others?

Yes

Reviewer Expertise:

Cognitive ergonomics

I confirm that I have read this submission and believe that I have an appropriate level of expertise to state that I do not consider it to be of an acceptable scientific standard, for reasons outlined above.
